# Triple-Negative Primary Breast Tumors Induce Supportive Premetastatic Changes in the Extracellular Matrix and Soluble Components of the Lung Microenvironment

**DOI:** 10.3390/cancers12010172

**Published:** 2020-01-10

**Authors:** Braeden Medeiros, David Goodale, Carl Postenka, Lori E. Lowes, Patti Kiser, Stephen Hearn, Nikki Salmond, Karla C. Williams, Alison L. Allan

**Affiliations:** 1 Department of Anatomy & Cell Biology, Western University, London, ON N6A 5W9, Canada; bmedeir4@uwo.ca; 2 London Regional Cancer Program, London Health Sciences Centre, London, ON N6A 5W9, Canada; david.goodale@lhsc.on.ca (D.G.); cpostenk@uwo.ca (C.P.); 3 London Regional Cancer Program and Flow Cytometry, London Health Sciences Centre, London, ON N6A 5W9, Canada; lori.lowes@lhsc.on.ca; 4 Department of Pathology & Laboratory Medicine, Western University, London, ON N6A 3K7, Canada; pkiser@uwo.ca; 5 Biotron Research Centre, Western University, London, ON N6A 3K7, Canada; hearnpatineur@hotmail.com; 6 Faculty of Pharmaceutical Sciences, University of British Columbia, Vancouver, BC V6T 1Z4, Canada; nikki.salmond@ubc.ca (N.S.); karla.williams@ubc.ca (K.C.W.)

**Keywords:** breast cancer, organ tropism, lung metastasis, breast cancer molecular subtype, extracellular vesicles (EVs)

## Abstract

The lung is one of the deadliest sites of breast cancer metastasis, particularly in patients with triple-negative (TN) disease. We hypothesized that the presence of a TN primary breast tumor induces changes in the extracellular matrix (ECM) and soluble components of the lung microenvironment that support metastatic behavior. SUM159 (TN) and MCF7 (luminal A) breast cancer cells were injected into mice, and primary breast tumors were established prior to assessing metastatic niche changes. We observed increased CD117^+^ hematopoietic progenitor cells in the bone marrow of SUM159 mice versus MCF7 or control mice (*p* < 0.05). Relative to mice bearing MCF7 tumors and non-tumor controls, mice bearing SUM159 tumors demonstrated enhanced expression of ECM proteins in the lung (fibronectin, tenascin-c and periostin), with similar changes observed in lung fibroblasts treated with extracellular vesicles (EVs) from TN breast cancer cells (*p* < 0.05). Exposure to lung-conditioned media (LCM) from SUM159 tumor-bearing mice resulted in increased migration/proliferation of both SUM159 and MCF7 cells relative to the control (*p* < 0.05). In contrast, LCM from MCF-7 tumor-bearing mice had no such effect. LCM from SUM159 tumor-bearing mice contained 16 unique proteins relative to other LCM conditions, including the metastasis-associated proteins CCL7, FGFR4, GM-CSF, MMP3, thrombospondin-1 and VEGF. These findings suggest for the first time that the TN breast cancer molecular subtype may be an important determinant of premetastatic changes to both the ECM and soluble components of the lung, potentially mediated via breast cancer-derived EVs.

## 1. Introduction

Breast cancer is the second most common cancer worldwide and the most common amongst women [1]. In both Canada and the United States, approximately 1 in 8 women will develop breast cancer over their lifetime. In 2019, there were a projected 1,762,450 new cases and 606,880 deaths from breast cancer in the United States [2]. Of these deaths, over 90% were attributed to metastasis-related complications that occur when breast cancer cells escape from the primary tumor and spread to distant organ sites [3]. Clinically, breast cancer is subdivided into four main molecular subtypes based on the expression of the estrogen receptor (ER), progesterone receptor (PR) and human epidermal growth factor receptor 2 (HER2), as well as the proliferative index (Ki67) [4]. These clinical molecular subtypes (in order of increasing aggressiveness) include luminal A (ER^+^/PR^+^/Ki67^−^), luminal B (ER^+^/PR^+^/HER2^−/+^/Ki67^+^), HER2-overexpressing (ER^−^/PR^−^/HER^+^) and basal-like/triple-negative (TN) (ER^−^/PR^−^/HER2^−^). Bone is the most predominant site of breast cancer metastasis overall (50.7%), followed by the lung (23.9%), liver (19.7%) and brain (5.7%) [5]. Patients with better prognosis subtypes such as luminal A/B have the highest propensity for bone metastasis, a disease state that is associated with increased bone fracture, bone pain and hypercalcemia [6]. In contrast, patients with more aggressive TN disease display an enhanced tendency to develop lung metastasis, often occurring within 5 years of the initial breast cancer diagnosis and leading to significant morbidity and mortality [7,8]. Of patients with lung metastases, 60–70% will succumb to their disease with a median survival of 25 months and will suffer significantly due to debilitating physiological effects [8]. This important clinical problem highlights the need to gain a greater understanding of the cellular and molecular processes that lead to lung metastasis in order to develop improved biomarkers and therapeutic strategies.

Pulmonary metastasis can be influenced by both the soluble and insoluble/stromal components of the lung. The soluble component is comprised of chemokines, cytokines, growth factors and soluble extracellular matrix (ECM) components, and we have previously identified several soluble factors derived from the normal physiological lung that promote TN breast cancer metastatic behaviors such as proliferation, migration and metastatic colonization [9]. The insoluble/stromal component of the lung is comprised primarily of ECM. In a malignant state, several of the proteins that form the ECM backbone (i.e., collagen, periostin, tenascin-c and fibronectin) are upregulated, promoting the recruitment of secondary cells that induce remodeling in preparation for metastasis [10,11,12,13,14]. Growing evidence in the literature suggests that the presence of a primary tumor can prime the insoluble/stromal lung microenvironment in order to create a more hospitable premetastatic niche for metastasis before tumor cells even arrive in the secondary organ. Elegant work by Lyden and colleagues first introduced the premetastatic niche concept in experimental models of melanoma and lung cancer [15], with further support from studies in preclinical models of pancreatic cancer [16]. This work has demonstrated that prior to the recruitment of cancer cells to distant organs, bone marrow-derived CD117^+^VEGFR1^+^ hematopoietic progenitor cells (HPCs) get recruited to the lung and induce ECM remodeling. The recruitment of these cells is mediated by the co-expression of the integrin VLA-4, which interacts with the ligand fibronectin. In a malignant state, CD117^+^ VEGFR1^+^ HPCs circulate and bind to areas of increased fibronectin deposition, inducing the release of the proteinase MMP9 and resulting in the breakdown of the basement membrane to promote recruitment and colonization of cancer cells [15]. Recent studies have suggested that these premetastatic changes at the secondary organ site may be mediated by extracellular vesicles (EVs) released by the primary tumor [16,17,18,19].

The importance of the lung metastatic niche in breast cancer and its relationship to clinically relevant prognostic features such as the molecular subtype remain poorly understood. In the current study, we tested the hypothesis that luminal A versus TN primary tumors would differentially induce changes in the stromal and soluble lung microenvironment, with more aggressive TN primary tumors demonstrating enhanced establishment of a supportive lung niche that promotes metastatic behavior. To investigate this, we used preclinical in vivo models in which luminal A (MCF7) or TN (SUM159) primary breast tumors were grown in female nude mice prior to investigation of potential premetastatic changes. Our results indicate that the presence of a SUM159 TN primary tumor results in enhanced production/mobilization of the CD117^+^ HPC population in the bone marrow and induces supportive premetastatic changes in both the stromal and soluble components of the lung. In contrast, luminal A MCF7 primary tumors were not able to induce these changes. In support of this, extracellular vesicles (EVs) isolated from different triple negative cell lines induced the expression of premetastatic factors in lung fibroblasts, while EVs from luminal a cell lines had no such effect. Taken together, these novel findings suggest for the first time that the TN breast cancer molecular subtype may be an important determinant of premetastatic changes to both the ECM and soluble components of the lung, potentially mediated via breast cancer-derived EVs.

## 2. Results

### 2.1. Mice Bearing Triple Negative SUM159 Primary Tumors Demonstrate an Increased CD117^+^ Population in the Bone Marrow 

**Figure 1:**
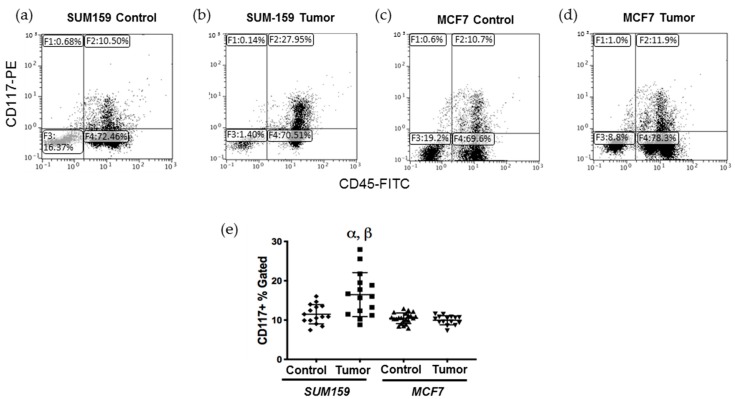
Mice bearing triple negative SUM159 tumors demonstrate an enhanced CD117^+^ cell population in the bone marrow. Triple-negative SUM159 or luminal A MCF7 human breast cancer cells were injected into the mammary fat pad of female nude mice, and primary breast tumors were allowed to develop up to a mean tumor size of 1500 mm^3^. Animals were euthanized at the endpoint, and bone marrow (BM) was immediately extracted and stained with primary anti-CD117-PE and anti-CD45-FITC antibodies and analyzed by flow cytometry as described in Supplemental Figure S2. Cells (10,000/sample) were analyzed to assess differences in CD117^+^ cells within the total BM population. (**a**–**d**) Representative flow cytometry histograms of the CD117^+^ BMDC population (gate F2) in (**a**) age-matched tumor-naïve mice (controls for SUM159); (**b**) SUM159 tumor-bearing mice; (**c**) age-matched tumor-naïve mice (controls for MCF7) and (**d**) MCF7 tumor-bearing mice. (**e**) Compiled flow cytometry data for all groups (*n* = 15 mice/group). Data are presented as the mean ± SEM. α = significantly different from respective age-matched controls. β = significantly different from MCF7 tumor-bearing mice (*p* < 0.05).

Previous studies have demonstrated that CD117^+^ bone marrow-derived cells (BMDCs) are produced/mobilized in the bone marrow in order to facilitate the induction of ECM remodeling in secondary organs prior to colonization by metastatic tumor cells [15]. To investigate this, bone marrow was isolated from tumor-naïve, age-matched control mice or mice bearing SUM159 or MCF7 mammary fat pad tumors grown to a mean tumor size of up to 1500 mm^3^ (Supplementary Figure S1), and the CD117^+^ BMDC population was analyzed using flow cytometry (Supplementary Figure S2a–c). We observed that mice bearing triple-negative SUM159 tumors had a significantly higher percentage of CD117^+^ cells in their bone marrow relative to mice bearing luminal A MCF7 tumors or tumor-naïve, age-matched control mice (*p* ≤ 0.05) (Figure 1). The percentage of CD117^+^ BMDCs did not correlate with either primary tumor size (Supplementary Figure S2d) or the length of time bearing a primary tumor (Supplementary Figure S2e).

### 2.2. Expression of Premetastatic Niche Markers Is Enhanced in the Lungs of Mice Bearing Triple Negative SUM159 Primary Tumors

**Figure 2:**
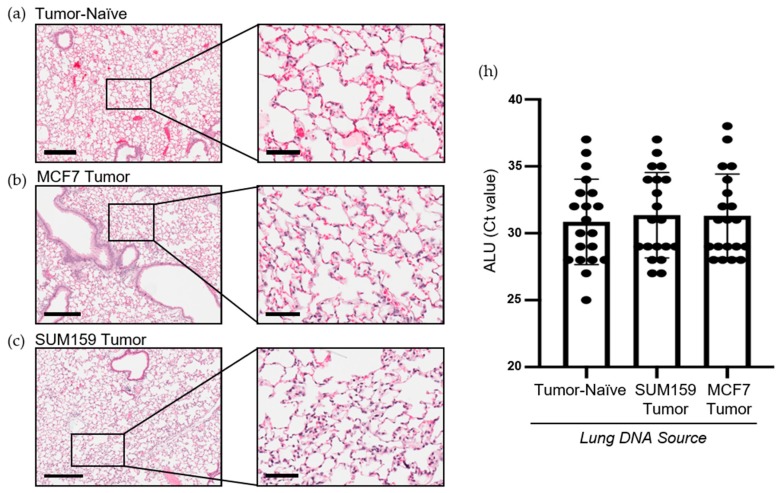

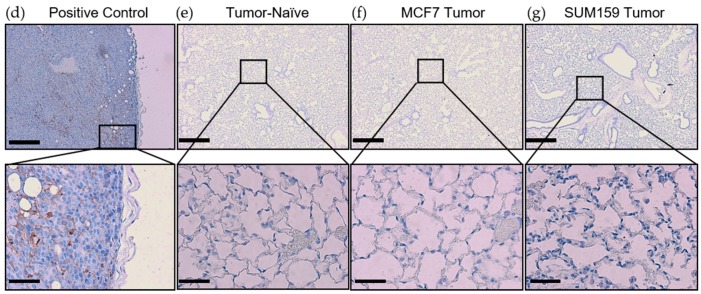
Mice bearing either SUM159 or MCF7 primary breast tumors had no evidence of lung metastasis at the time of the endpoint/analysis**.** Triple-negative SUM159 or luminal A MCF7 human breast cancer cells were injected into the mammary fat pad of female nude mice, and primary breast tumors were allowed to develop up to a mean tumor size of 1500 mm^3^. Animals were euthanized at the endpoint, and lungs were harvested and either formalin-fixed or snap-frozen. (**a**–**g**) Formalin-fixed, paraffin-embedded tissues were sectioned (4 µm) and stained with (**a**–**c**) hematoxylin and eosin (H&E) or (**e**–**g**) subjected to immunohistochemistry with a human-specific mitochondrial cytochrome C oxidase antibody prior to analysis by a trained veterinary pathologist (P.K.). Four random sections/organ/mouse and 10 high-powered fields of view (FOVs)/section were analyzed at 400× magnification for the presence of metastatic tumor cells (*n* = 9 mice/group). Representative lung sections are shown from (**a**,**e**) tumor-naïve control mice; (**b**,**f**) MCF7 tumor-bearing mice and (**c**,**g**) SUM159 tumor-bearing mice. (**d**) Positive control; MCF7 primary human breast tumor tissue. Scale bars: low magnification images = 300 μm (left panels *(***a**–**c***)* and top panels *(***d**–**g**) and high magnification images = 70 μm (right panels *(***a**–**c***)* and bottom panels *(***d**–**g**). (**h**) DNA was isolated from snap-frozen lung tissue from tumor-naïve control mice, MCF7 tumor-bearing mice and SUM159 tumor-bearing mice (*n* = 15/group) and subjected to qPCR analysis using primers specific to the human ALU sequence.

Prior to cancer cell seeding at the secondary site, the organ must become competent to enable metastatic tumor growth. The ECM and soluble components of the lung are crucial for mediating this switch to a competent state [15]. To investigate differences in premetastatic niche markers, lungs were harvested at the endpoint from mice bearing SUM159 or MCF7 primary tumors and compared to lungs from corresponding tumor-naïve, age-matched control mice using histopathological and immunohistochemical analysis. Histopathological analysis demonstrated that mice bearing either SUM159 or MCF7 primary tumors had not yet developed lung metastasis at the time of the endpoint/analysis (Figure 2a–c). The absence of metasasis was also confirmed via immunohistochemical staining of additional lung sections with a human-specific mitochondrial cytochrome C oxidase antibody (Figure 2d–g) and by qPCR analysis of DNA isolated from the lungs of mice using primers specific to the human ALU sequence (Figure 2h). 

**Figure 3:**
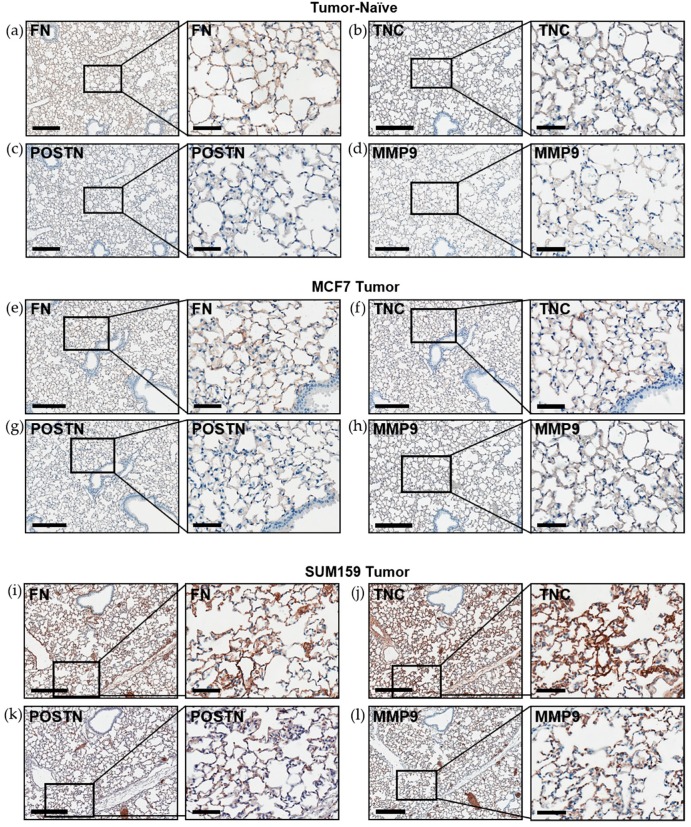
Expression of the premetastatic niche markers fibronectin, tenascin-c, periostin and MMP9 is enhanced in the lungs of mice bearing triple-negative SUM159 primary tumors. Triple-negative SUM159 or luminal A MCF7 human breast cancer cells were injected into the mammary fat pad of female nude mice, and primary breast tumors were allowed to develop up to a mean tumor size of 1500 mm^3^. Animals were euthanized at the endpoint, lungs were harvested and formalin-fixed and paraffin-embedded tissues were sectioned (4 µm) and subjected to immunohistochemical staining with antibodies against murine fibronectin (FN) (**a**,**e**,**i**); tenascin-c (TNC) (**b**,**f**,**j**); periostin (POSTN) (**c**,**g**,**k**) or MMP9 (**d**,**h**,**l**) prior to analysis by a trained veterinary pathologist (P.K.). Four random sections/lung/mouse and 10 high-powered fields of view (FOVs)/section were analyzed at 400× magnification (*n* = 9 mice/group). Representative lung sections are shown from (**a**–**d**) tumor-naïve control mice; (**e**–**h**) MCF7 tumor-bearing mice and (**i**–**l**) SUM159 tumor-bearing mice. Scale bars: low magnification images = 300 μm (left panels); high magnification images = 70 μm (right panels).

**Figure 4:**
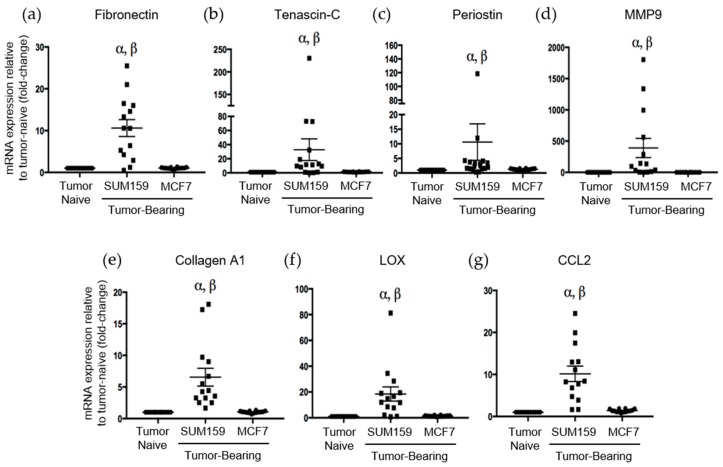
Mice bearing triple negative SUM159 tumors demonstrate increased mRNA expression of the premetastatic markers fibronection, tenascin-c, periostin, MMP9, collagen A1, LOX and CCL2 in the lungs. Triple-negative SUM159 or luminal A MCF7 human breast cancer cells were injected into the mammary fat pad of female nude mice, and primary breast tumors were allowed to develop up to a mean tumor size of 1500 mm^3^. Animals were euthanized at the endpoint, and lungs were harvested and snap-frozen. RNA was isolated from lungs and subjected to qRT-PCR analysis to assess the expression of murine fibronectin (**a**), tenascin-c (**b**), periostin (**c**), MMP9 (**d**), collegen A1 (**e**), LOX (**f**) and CCL2 (**g**) (*n* = 15 mice/group). Data are presented as the mean ± SEM. α = significantly different from age-matched tumor-naïve controls. β = significantly different from MCF7 tumor-bearing mice (*p* < 0.005).

Immunohistochemical analysis of premetastatic niche markers indicated that, relative to mice bearing luminal A MCF7 primary tumors or age-matched tumor-naïve controls, mice bearing triple-negative SUM159 primary tumors demonstrated enhanced expression of fibronectin, tenascin-c, periostin and MMP9 in the lung (Figure 3). To quantify these observations, RNA was isolated from the lungs of tumor-bearing or tumor-naïve mice and analyzed by qRT-PCR. Consistent with the IHC results, we observed that lungs from mice bearing triple-negative SUM159 primary tumors showed increased expression of murine fibronectin, tenascin-c, periostin, MMP9 and collagen A1 compared to lungs from mice bearing luminal A MCF7 primary tumors or age-matched tumor-naïve mice (*p* ≤ 0.05) (Figure 4a–e). The expression of lysyl oxidase (LOX) and CCL2 was also examined. Expression of LOX has been shown to be increased in the premetastatic organ microenvironment, inducing collagen crosslinking and promoting the recruitment of BMDCs for ECM remodeling [20]. Coupled with collagen crosslinking, CCL2 acts as a strong attractant for BMDCs cells. We observed a significant increase in LOX and CCL2 expression in the lungs of mice bearing triple-negative SUM159 breast tumors relative to lungs from mice bearing luminal A MCF7 tumors or tumor-naïve mice (Figure 4f,g). Taken together, these results indicate that the presence of a triple-negative SUM159 primary tumor induces the development of premetastatic niche characteristics in the lung by enhancing the expression of ECM proteins and effector molecules that aid in the recruitment of BMDCs. 

### 2.3. The Presence of a Triple-Negative SUM159 Breast Tumor Modifies the Soluble Lung Microenvironment to Enhance Breast Cancer Cell Proliferation and Migration 

**Figure 5:**
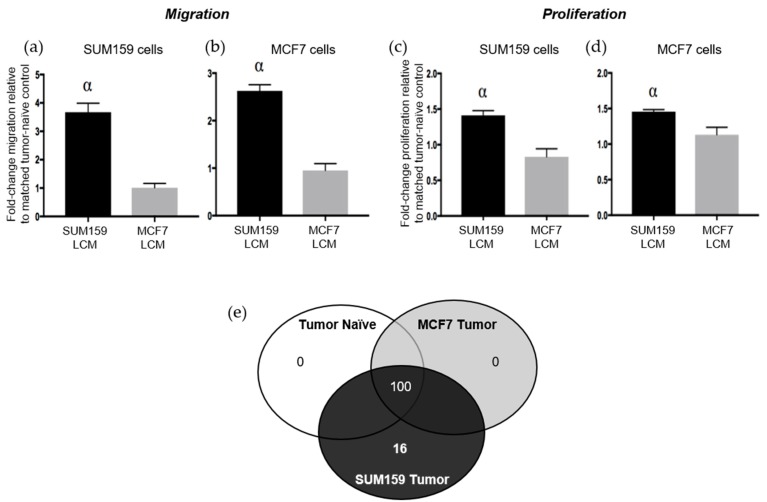
The presence of a triple-negative SUM159 breast tumor modifies the soluble lung microenvironment to enhance breast cancer cell migration and proliferation. Triple-negative SUM159 or luminal A MCF7 human breast cancer cells were injected into the mammary fat pad of female nude mice, and primary breast tumors were allowed to develop up to a mean tumor size of 1500 mm^3^. Animals were euthanized at the endpoint, and lungs were harvested and used for the generation of lung-conditioned media (LCM) as described previously [9]. To assess migration, (**a**) SUM159 or (**b**) MCF7 cells were subjected to transwell migration assays for 18 h as described in the Materials & Methods. To assess proliferation, (**c**) SUM159 or (**d**) MCF7 cells were subjected to BrdU incorporation assays as described in the Materials & Methods. Five high-powered fields of view (HP-FOV) were used to enumerate migrated or proliferating cells using ImageJ software (NIH). Data are presented as the mean ± SEM (LCM from *n* = 15/mice per group). α = significantly different from LCM from MCF7 tumor-bearing mice (*p* < 0.05). (**e**) RayBio^®^ Mouse Antibody Array AAM-BLM-1 membranes (RayBiotech; *n* = 3/group) were exposed to dialyzed, biotin-labeled media samples, washed, labeled with HRP-streptavidin and visualized using chemiluminescence and film exposure. Venn diagram showing the number of similar versus unique proteins identified under each LCM condition.

The results presented above suggest that the presence of a triple negative SUM159 primary breast tumor can induce premetastatic changes in the lung ECM relative to the presence of a luminal A MCF7 primary tumor or no tumor. We have previously observed that soluble factors derived from the normal physiological lung in tumor-naïve mice can promote TN breast cancer proliferation and migration [9]. We were therefore also interested in evaluating whether the presence of a TN primary tumor would further enhance the ability of the soluble lung microenvironment to support metastatic behavior. Lung-conditioned media (LCM) were generated from the lungs of mice bearing SUM159 or MCF7 breast tumors and compared to LCM generated from the lungs of age-matched tumor-naïve mice. Breast cancer cells were exposed to LCM using a matched or cross-over design and assessed for changes in breast cancer cell migration and proliferation. Relative to LCM from age-matched tumor-naïve mice, we observed that LCM generated from the lungs of mice bearing TN SUM159 primary tumors promoted the migration (Figure 5a,b) and proliferation (Figure 5c,d), not only of matched SUM159 cells, but also of luminal A MCF7 breast cancer cells in cross-over experiments (*p* < 0.05). In contrast, exposure to LCM generated from the lungs of MCF7 tumor-bearing mice did not result in any significant differences in proliferation or migration in either cell line compared to LCM from control mice. 

To begin to elucidate the molecular basis for these observations, LCM from both tumor-bearing and tumor-naïve mice was subjected to protein array analysis to interrogate changes in the presence of chemokines, cytokines, growth factors and soluble ECM factors between experimental groups (Supplementary Table S1). Interestingly, we observed that the complement of soluble proteins produced by the lungs of mice bearing luminal A MCF7 primary tumors was identical to that of lungs from tumor-naïve mice, with 100 proteins reproducibly identified across 3 replicate experiments (Figure 5e). In contrast, 16 different proteins were identified to be consistently present and unique in LCM generated from the lungs of mice bearing triple-negative SUM159 primary tumors (Supplementary Figure S3, Supplementary Table S2). Of these, 6 proteins have previously been demonstrated to mediate lung metastasis (Table 1), including CCL7 [21,22], FGFR4 [23,24], GM-CSF [25], MMP3 [26], thrombospondin-1 [27] and VEGF [28,29]. Taken together, these results suggest for the first time that, in addition to helping to establish a premetastatic niche through modification of the lung ECM, the presence of a triple-negative SUM159 tumor also induces changes in the soluble lung microenvironment that are supportive of breast cancer metastatic behavior.

### 2.4. Extracellular Vesicles (EVs) from Triple Negative Breast Cancer Cells Induce the Expression of the Premetasatic ECM Markers Periostin and Fibronectin in Lung Fibroblasts 

Finally, we wanted to begin to elucidate the potential mechanisms by which these premetastatic changes in the lung may be occurring and to expand our investigations to additional TN and luminal A models. To do this, we focused on the possible role of breast cancer-derived extracellular vesicles (EVs). Although EVs were originally considered waste removal mechanisms of cells, a strong body of evidence has now demonstrated that EVs contain functional cargos of proteins, nucleic acids and lipids that facilitate cell–cell communication [30]. Tumor-derived EVs have been shown to be important mediators of metastasis with the ability to target specific organs and to be taken up by recipient cells in order to induce stromal remodeling, alter soluble factor secretion profiles and stimulate angiogenesis [17,18,19]. However, the role of EVs in inducing premetastatic changes in the lung in the context of the breast cancer molecular subtype has yet to be investigated.

**Figure 6:**
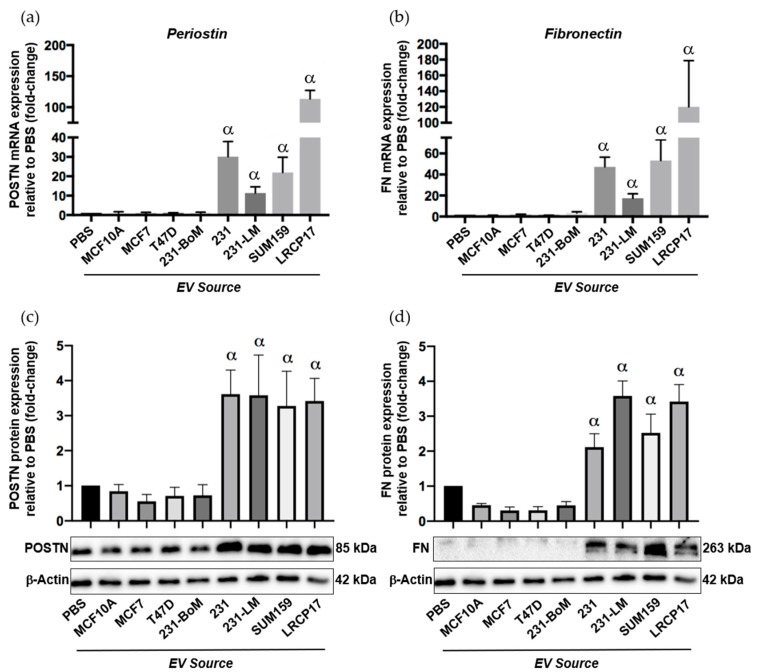
Extracellular vesicles (EVs) from triple negative breast cancer cells induce expression of the premetasatic ECM markers periostin and fibronectin in lung fibroblasts. Breast cancer-derived EVs were isolated from multiple different human breast cancer cell lines as described in the Materials and Methods and characterized as described in Supplemental Figure S4. Cell lines included non-tumorigenic control cells (MCF10A), two luminal A cell lines (MCF7, T47D), three TN cell lines (MDA-MB-231 (231), SUM159 and LRCP17 (derived from a patient-derived xenograft model)). As additional controls, we also included lung-seeking 231-LM and bone-seeking 231-BoM cells. Primary normal human lung fibroblasts (NHLFs) were treated with 50 µg of EVs from each cell line source every 12 h for 48 h prior to RNA and protein isolation. (**a**,**b**) mRNA expression of periostin (**a**) and fibronectin (**b**) in NHLFs treated with breast cancer-derived EVs. (**c**,**d**) Protein expression of periostin (**c**) and fibronectin (**d**) in NHLFs treated with breast cancer-derived EVs, including densitometric analysis (*n* = 3) (top) and representative immunoblots (bottom). β-Actin was used as a loading control. Data are presented as the mean ± SEM; fold-change in expression relative to the PBS control. α = significantly different from the PBS control (*p* < 0.05).

To begin to assess this, we isolated EVs from multiple different human breast cancer cell lines including non-tumorigenic control cells (MCF10A), two luminal A cell lines (MCF7, T47D) and three TN cell lines (SUM159, MDA-MB-231 and LRCP17 (a cell line derived from a TN patient-derived xenograft model)). As additional controls, we also included lung-seeking 231-LM cells [31], which we expect to show similar expression patterns to the other TN cell lines, and bone-seeking 231-BoM cells [32], which we expect to show a similar expression pattern to luminal A cells based on their similar preference for bone versus lung. Isolated EVs were characterized using transmission electron microscopy (TEM) (Supplementary Figure S4a) and nanoparticle tracking analysis (NTA), which confirmed that the isolated EV population was between 38–483 nm (Supplementary Figure S4b). Immunoblotting confirmed the presence of specific EV proteins (CD63, TSG101, CD9; [33]) (Supplementary Figure S4c). Validated EVs were then labeled with the green fluorescent membrane dye PKH67 and incubated with primary normal human lung fibroblasts to evaluate the influence of different breast cancer-derived EVs on the expression of premetasatic niche markers (Supplementary Figure S4d). In support of our in vivo findings, we observed that EVs from the TN cell lines MDA-MB-231, SUM159 and LRCP17 induced the expression of the premetastatic niche markers periostin and fibronectin in lung fibroblasts at both the RNA (Figure 6a,b) and protein (Figure 6c,d) levels (*p* < 0.05). In contrast, EVs from luminal A (MC7 and T47D) cell lines had no significant effect on the expression of periostin and fibronectin. Using multiple cell lines, these results suggest a potential mechanism for the observed differential lung ECM changes seen in our in vivo experiments. 

## 3. Discussion

The majority of breast cancer deaths are attributed to metastasis-related complications [3]. Clinical studies have demonstrated that breast cancer preferentially metastasizes to the brain, bone, liver, lung and lymph nodes, with variable organ tropism across molecular subtypes [5]. However, it remains unclear how and why this observed organ tropism occurs. Of these sites, lung metastasis is associated with significant morbidity and mortality, with no effective way of predicting lung tropism or detecting it early [6]. 

Interestingly, the lung is the first major capillary bed that a breast cancer cell encounters after escaping from the primary breast tumor into the vasculature and being subjected to normal physiological blood flow patterns [34]. As these cancer cells transverse through the capillaries, they come into contact with up to 100 m^2^ of surface vasculature. Coupled with the fact the cancer cells are approximately five times the diameter of the pulmonary capillaries, the probability of breast cancer cells arresting and extravasating into lung tissue is exceedingly high [34,35]. Previous work by Luzzi et al. [36] demonstrated that the process of metastasis up to and including the extravasation step is quite efficient. Despite this, once cancer cells are physically delivered to the secondary organ, only ~0.01% of these cells are able to successfully initiate and sustain growth in the secondary site in order to generate clinically relevant macrometastases. This highlights the critical need for the microenvironment at the secondary site to be suitable for initiating and sustaining cancer growth. However, the details of how and when the lung environment becomes favorable for metastatic growth has yet to be elucidated in the context of breast cancer molecular subtype. Several studies have demonstrated that the presence of a primary tumor has the potential to prime the lung in order to produce a supportive environment for secondary cancer cell seeding and growth, known as the premetastatic niche [15,16,20]. The current study suggests for the first time that breast cancer molecular subtype of the primary tumor (TN versus luminal A) may be an important potential determinant of premetastatic niche formation in the lung through modification of both ECM and soluble components of the lung microenvironment.

We observed that, compared to mice bearing luminal A MCF7 tumors or age-matched controls, premetastatic ECM characteristics including expression of fibronectin, tenascin-c, periostin and collagen A1 were enhanced in the lungs of mice bearing triple-negative SUM159 tumors. These proteins form a complex network that coordinates ECM-modulated signal transduction and adhesion at the secondary site [10,11,12,13,14,15,16]. Fibronectin has been demonstrated to promote cancer cell migration and invasion in the lung with the potential to confer resistance to therapy [13]. The expression of fibronectin regulates the expression of several crucial pro-metastatic proteins, such as MMP9 [37]. MMP9 expression has been shown to promote tumor cell invasion in the lung and recruitment of BMDCs to further induce pro-metastatic ECM remodeling [38]. Beyond modulating expression, ECM proteins are crucial in maintaining the complex architecture of the matrix. The matricellular protein periostin has been shown to promote the incorporation of tenascin-c into the ECM [39]. Periostin acts as a bridge using adjacent domains to interact with tenascin-c and other ECM proteins such as fibronectin and collagen. This relationship between periostin and tenascin-c is linked to their association with lung metastasis and promotion of cancer cell migration, proliferation and invasion [10]. We also observed that expression of collagen A1 was increased in the lungs of mice bearing TN SUM159 primary tumors relative to those bearing luminal A MCF7 primary tumors or tumor-naïve mice. Interestingly, this was accompanied by increased LOX and CCL2 expression. LOX mediates the crosslinking of collagen, and in addition to the expression of CCL2, recruits BMDCs to the lung to generate a pro-metastatic environment [20].

Beyond recruiting metastatic cancer cells, fibronectin has been demonstrated to act as a strong attractant for bone marrow-derived cells (BMDCs), specifically those expressing CD117, VEGFR1 and VLA-4. Previous studies have shown that CD117^+^ BMDCs are recruited to the lung (specifically to regions of increased fibronectin) prior to the arrival of metastatic cancer cells [15]. In the current study, mice bearing TN SUM159 primary tumors showed a significant increase in the CD117^+^ population in the bone marrow relative to age-matched controls or MCF7 tumor-bearing mice, suggesting that the TN molecular subtype may influence this subset of the bone marrow population and mobilize it in preparation for metastasis. 

In addition to determining how the presence of a TN primary breast tumor influences the lung ECM, we also investigated the effect on the soluble lung microenvironment. The lung produces soluble factors (cytokines, chemokines, growth factors and soluble ECM proteins) which are crucial for mediating pulmonary metastasis [40]. Previous work by our laboratory has demonstrated that the normal physiological lung in tumor naïve mice produces various soluble factors that promote metastatic behavior [9]; however, the ability of the primary tumor to further enhance the soluble lung microenvironment in preparation for metastasis has not been investigated. To assess this, lung-conditioned media (LCM) were generated from mice bearing TN/luminal primary tumors and tumor-naïve control mice. Exposure of SUM159 or MCF7 breast cancer cells to LCM from SUM159 tumor-bearing mice resulted in increased breast cancer proliferation and migration compared to LCM from MCF7 tumor-bearing mice or tumor-naïve mice. This indicates that the composition of the soluble lung microenvironment has been altered in a primary tumor-dependent manner. To begin to elucidate the molecular basis for these observations, a protein array was used and identified the presence of 16 unique factors in the LCM of mice bearing TN tumors, including 6 that have been associated with lung metastasis, inflammation, immunosuppression and angiogenesis (CCL7, FGFR4, MMP3, thrombospondin-1, VEGF and GM-CSF) [21,22,23,24,25,26,27,28,29]. A limitation of this protein array is its ability to only interrogate 308 soluble factors, a small fraction of the lung secretome. Future studies are needed to expand on these results using more sensitive and unbiased methods such as mass spectrometry, which may enable the identification of how the soluble component changes in a tumor-bearing state and further differentiate differences in a molecular subtype-specific manner.

Taken together, the in vivo changes to the stromal and soluble components of the lung suggest that there may be unique communication between the primary tumor and the lung that is different between the TN SUM159 breast cancer model and the luminal A MCF7 model. To begin to investigate this, we focused our attention on extracellular vesicles (EVs) since previous studies have identified EVs as mediators of organ tropism and premetastatic niche formation in the lung [17,41,42]. To evaluate this, the expression of the premetastatic niche markers periostin and fibronectin was evaluated in normal human lung fibroblasts following treatment with EVs from several different TN and luminal A breast cancer cell lines. In support of our in vivo findings, we observed that treatment of lung fibroblasts by TN EVs significantly increased periostin and fibronectin expression relative to EVs from luminal A or control breast cancer cells. These results suggest the intriguing possibility that EVs produced by TN breast cancer cells may preferentially signal the lung microenvironment in preparation for successful metastasis. However, further investigations are required to elucidate the molecular differences in EV cargo (i.e., DNA, RNA and protein content) and the subsequent functional and mechanistic implications for breast cancer metastasis in the lung. Given the clinical disparity between sites of metastasis and overall survival between luminal A and TN subtypes of breast cancer [4,5], the ability to identify unique EV profiles may be useful for both understanding breast cancer biology and developing new clinical biomarkers for lung metastasis in the future. 

In summary, the novel findings presented in this study demonstrate that the presence of triple-negative SUM159 versus luminal A MCF7 primary breast tumors induces differential changes in both the ECM and soluble lung microenvironment, with more aggressive triple-negative SUM159 primary tumors demonstrating enhanced establishment of a supportive lung niche that promotes metastatic behavior. These findings support the concept that the molecular subtype of the primary tumor may influence the ability of the lung to be “primed” in preparation for metastasis. The luminal A and TN molecular subtypes were chosen for initial investigation because they are on the opposite ends of the spectrum of aggressiveness and display a separation clinically with regards to the propensity for developing lung metastasis. While the molecular subtype is likely not the only factor that influences premetastatic changes to the stromal and soluble lung microenvironment in breast cancer, the differential results between TN and luminal A models seen in this study set the stage for future studies aimed at elucidating this concept further through investigation of other clinically used molecular subtypes (HER2^+^ and luminal B), as well as subsets of the triple-negative subtype. 

## 4. Materials and Methods 

### 4.1. Cell Culture 

The SUM-159 human triple negative cell line was obtained from Asterand Inc. (Detroit, MI, USA) and was cultured in HAMS:F12 + 5% fetal bovine serum (FBS), 0.5% insulin, 0.1% hydrocortisone 1% HEPES. The MCF7 luminal A human breast cancer cell line was obtained from American Type Culture Collection (ATCC; Manassas, VA, USA) and was cultured in Dulbecco’s Modified Eagle’s Medium (DMEM; Invitrogen; Carlsbad, CA, USA) + 10% FBS. Cell lines were authenticated via third party testing (IDEXX BioAnalytics, Columbia, MO, USA) between October 2018 and October 2019. The MCF10A cell line was obtained from ATCC and was cultured in DMEM:F12 + 5% FBS + 100 ng/mL cholera toxin. The MDA-MB-231 cell line was obtained from Dr. Ann Chambers (London Health Science Centre, London, Canada) and was cultured in DMEM:F12 + 10% FBS. The MDA-MB-231-4175 LM2 (231-LM; lung-seeking metastatic variant [31]) and MDA-MB-231-1833 BoM (231-BoM; bone-seeking metastatic variant [32]) cell lines were obtained from Dr. Joan Massagué (Memorial Sloan Kettering Cancer Center, New York, NY, USA) and were cultured in DMEM + 10% FBS. The LRCP17 cell line was generated in-house from a TN patient-derived xenograft (PDX) model after being grown as a mammary fat pad tumor in NOD/SCID mice for 2 passages, enzymatically dissociated and established in culture in DMEM:F12 + 10% FBS + 0.5% insulin, 0.1% hydrocortisone, 1% HEPES and 0.1% BSA. The original breast tumor biopsy used to generate the LRCP17 PDX was obtained from a breast cancer patient with metaplastic TN (ER^−^PR^−^HER2^−^) breast cancer following informed consent under a human ethics protocol approved by the University of Western Ontario HSREB (#103613). Primary normal human lung fibroblasts (NHLFs) were obtained from Lonza (Basel, Switzerland) and were cultured in FGM-2 fibroblast growth medium (Lonza) that included supplementation with 0.5% insulin, 0.1% human basic fibroblast growth factor (hbFGF), 0.1% gentamicin/ amphotericin (GA-1000) and 2% FBS. 

### 4.2. In Vivo Studies 

Animal experiments were carried out in accordance with the Canadian Council of Animal Care under a protocol approved by the University of Western Ontario Animal Care Committee (#2017-136). MCF7 and SUM159 human breast cancer cells were suspended in Hanks’ Balanced Salt Solution (HBSS) (Sigma, Kawasaki, Japan) at a concentration of 1 × 10^7^ cells/mL. Cell suspensions (100 μL; 1 × 10^6^ cells/mouse) were injected into the mammary fad pad (m.f.p.) of 6–8-week old female nude mice (Athymic Nude-Foxn1nu; Envigo, Mississauga, ON, Canada) (*n* = 54 mice/group) as described previously [43]. Mice injected with MCF7 cells or their matched tumor-naïve controls were implanted with subcutaneous time-release estrogen pellets (0.10 mg/pellet) with 90-day release (Innovative Research of America, Sarasota, FL, USA) for the duration of the experiments. Primary tumor size was longitudinally assessed using weekly digital caliper measurements in 2 perpendicular dimensions and was calculated using the formula: volume = 0.52 × (width)^2^ × (length). Primary tumors were allowed to grow up to 1500 mm^3^, with mice in tumor-bearing groups (SUM159/MCF7) sacrificed at the same time as age-matched, tumor-naïve control mice. Within each group, mice were randomly assigned to 4 subgroups for different tissue uses: for *n* = 15 mice, lungs were flash frozen for DNA/RNA isolation; for *n* = 15 mice, lungs were collected for conditioned media isolation and for *n* = 9 mice, lungs were formalin fixed for histopathology. Additional tissues (primary tumors, lymph nodes, liver, bone, brain) were formalin-fixed for histology, and bone marrow was collected and used for flow cytometry analysis (*n* = 15). 

### 4.3. Flow Cytometry Analysis

At the endpoint, bone marrow was extracted from the femur and tibia of mice, and a cell suspension of bone marrow (BM) was obtained. Red blood cells were lysed with NH_4_Cl for 10 min prior to incubation with 20 μL of phycoerythrin (PE)-conjugated anti-mouse CD117 antibody (StemCell Technologies, Vancouver, BC, Canada) and 10 μL of fluorescein (FITC)-conjugated anti-mouse CD45 antibody (BD Biosciences, Mississauga, ON, Canada). Cells were washed with PBS and resuspended in 500 μL of flow buffer (5% FBS + 0.5% EDTA). All samples were stored on ice in the dark and analyzed on a FC500 flow cytometer (Beckman Coulter, Miami, FL, USA).

### 4.4. Histopathology and Immunohistochemistry (IHC) 

Lungs isolated from mice were formalin-fixed (10%), paraffin-embedded and sectioned (4 µm) prior to staining with hematoxylin and eosin (H&E) or for use in immunohistochemical analysis. For IHC, samples were deparaffinized with xylene and were successively washed with a gradient of ethanol washes (100–70%). Antigen retrieval was subsequently performed, samples were incubated in a 100 °C water bath immersed in citrate buffer (50 mM of citric acid, pH 6.0) for 20 min and cooled at room temperature. Slides were stained using an IHC kit (Cat#: ab64264, Abcam, Cambridge, UK) using antibodies detailed in Supplementary Table S3, diluted in 5% BSA and incubated for 1 h at room temperature. Nuclei were stained with hematoxylin. Samples were analyzed by a trained veterinary pathologist (P.K.) using four random sections/organ/mouse and 10 high-powered fields of view (HP-FOVs)/sections at 400× magnification (*n* = 9 mice/group).

### 4.5. Quantitative PCR (qPCR) and Quantitative RT-PCR (qRT-PCR)

Quantitative qPCR for the human ALU sequence was carried out as previously described [44]. Briefly, lungs were homogenized, and DNA was isolated using a DNA purification kit (Cat#: 69504, Qiagen; Hilden, Germany). Relative quantification of the presence of the human ALU sequence was performed using SYBR Green Mastermix (Invitrogen) and the human ALU primers 5′-GTCAGGAGATCGAGACCATCCT-3′ (forward) and 5′-AGTGGCGCAATCTCGGC-3′ (reverse) as previously described [44].

For qRT-PCR analysis of homogenized lung tissue from in vivo experiments or normal human lung fibroblasts (NHLFs), TRIzol (Invitrogen) was used to isolate total RNA followed by purification using an RNA purification kit (Cat#: 12183555, Invitrogen). Total RNA (1 μg) was reverse-transcribed using Superscript IV VILO Master Mix (Invitrogen) and the Eppendorf Mastercycler Gradient (Eppendorf, Hamburg, Germany). Relative quantification of RNA expression of murine periostin, tenascin-c, fibronectin, MMP9, collagen A1, LOX, CCL2 (lung tissue) or human periostin and fibronectin (NHLFs) was determined by quantitative PCR using Taqman Fast Advanced Mastermix (Invitrogen) and Taqman primers detailed in Supplementary Table S4. Relative RNA expression was determined using the 2^−ΔΔCT^ method as previously described, with GAPDH used for normalization [45]. 

### 4.6. Generation of Lung-Conditioned Media

Lung-conditioned media (LCM) were generated as described previously [9]. Briefly, at the time of sacrifice, lungs were aseptically removed, washed and kept in cold sterile PBS on ice. Lungs were weight normalized by resuspension at a 4:1 media to tissue (vol/wt) ratio in DMEM:F12 + 1× MITO^+^ (BD Biosciences, Mississauga, Ontario) + penicillin (50 U/mL)/streptomycin (50 μg/mL) (pen/strep; Invitrogen). Lungs were cultured for 24 h; LCM were collected, filtered through 0.22 μm filters to remove cellular debris and stored at −80 °C. To account for mouse-to-mouse variability, LCM from multiple mice were pooled before use in functional experiments.

### 4.7. Breast Cancer Cell Migration and Proliferation Assays

Differences in migration between MCF7 and SUM159 cells in response to LCM were assessed using transwell migration assays. Transwell inserts (8 µm pore size) were coated with gelatin and exposed to media in the bottom well, including LCM from tumor-bearing mice (MCF7 or SUM159), LCM from corresponding age-matched control mice or basal media (DMEM:F12 + 1X MITO^+^). Breast cancer cells (5 × 10^4^ cells/well) were seeded onto the top portion of each transwell chamber and incubated for 18 h at 37 °C with 5% CO_2_ prior to staining and assessment of differences in migration. Differences in proliferation between MCF7 and SUM159 cells in response to LCM were assessed by BrdU incorporation. Breast cancer cells (1 × 10^5^ cells/well) were plated in chamber slides and incubated for 24 h at 37 °C and 5% CO_2_ with basal media + 10% FBS. Media were replaced with basal media, and cells were incubated for 5 days at 37 °C, 5% CO_2_. The basal medium was then replaced with LCM from tumor-bearing mice (MCF7 or SUM159), LCM from corresponding age-matched control mice or basal medium for 24 h. Cells were formalin-fixed and incubated with mouse anti-human BrdU primary antibody (Invitrogen) for 12 h followed by incubation with a goat anti-mouse Alexa488 (Invitrogen) secondary antibody and DAPI. Five HP-FOVs were analyzed for each well, and a mean number of migrated or proliferating cells/FOV was calculated using ImageJ software (NIH, version 1.51(100), Bethesda, MD, USA). 

### 4.8. Protein Array Analysis

To assess similarities and differences in soluble factors present under different LCM conditions, RayBio AAM-BLM-1 label-based mouse antibody arrays were used to simultaneously assess the expression of 308 soluble murine target proteins (RayBiotech Inc, Norcross, GA, USA). Post-dialysis protein concentration of LCM (*n* = 3 per condition) was determined using the DC protein assay (Bio-Rad Laboratories, Mississauga, ON, Canada), labeled and incubated with protein arrays as per manufacturer’s instructions. Results were visualized using chemiluminescence and film exposure (CL-XPosure Film; Pierce, Thermo Fisher Scientific, Waltham, MA, USA). Results (*n* = 3 per media condition) were analyzed using the RayBiotech analysis tool for AAM-BLM-1. Sixteen confirmed protein hits unique to LCM generated from the lungs of mice bearing SUM159 tumors were identified as having values > 1 after background subtraction and validation across three replicates. Due to differences in antibody affinities for target antigens, quantitative comparison between different proteins was not feasible using this platform. 

### 4.9. Isolation and Characterization of Breast Cancer-Derived Extracellular Vesicles (EVs)

#### 4.9.1. EV Isolation 

Human breast cancer cells (MCF10A, T47D, MCF7, SUM159, MDA-MB-231, 231-LM, 231-BoM and LRCP17) were grown to approximately 80% confluency in normal growth media. Culture media were replaced with serum-free media, and cells were incubated under hypoxic culture conditions (37 °C, 1% CO_2_) for 48 h to enhance EV production and packaging [46,47]. Culture media were harvested, and EVs were isolated as previously described [48]. Briefly, cells and debris were cleared from the harvested culture media by centrifugation (30 min 1000× *g*), followed by filtration using 0.22 μm filters (Millipore; Billerica, MA) and removal of larger vesicles using an additional centrifugation step (1 h 13,000× *g* at 4 °C). Cell-free media were concentrated by ultrafiltration using Centricon Plus-70 centrifugal filters (100 kDa; Millipore) and centrifuged at 1000× *g* at 4 °C. EVs were subsequently purified by overlaying concentrated samples on qEV size-exclusion chromatography columns (Izon Science Ltd.; Christchurch, New Zealand) followed by elution with PBS. Finally, the eluates from the qEV columns were concentrated using Amicon Ultra-4 10 kDa nominal molecular weight centrifugal filter units (Millipore) to a final volume of approximately 200 µL. Following EV characterization by transmission electron microscopy (TEM), nanoparticle tracking analysis (NTA) and immunoblotting (described below), concentrated EVs were labelled with the green fluorescent membrane dye PKH67 and used to treat normal human lung fibroblasts (NHLFs) prior to analysis by immunoblotting (50 µg EVs every 12 h for 48 h per 60 mm plate of NHLFs at 60–70% confluency; *n* = 3 replicates per EV treatment).

#### 4.9.2. EV Characterization by TEM

Analysis of EVs by TEM was carried out as previously described [49,50]. After EV isolation, 5 μL of the final suspension was absorbed onto freshly prepared carbon-coated 400 mesh nickel TEM grids and negative staining was done using 1% uranyl acetate in 1% aqueous methyl cellulose. Images were collected using a Philips 420 transmission electron microscope equipped with an AMT 4K megapixel XR41S-B camera (Thermo Fisher Scientific). 

#### 4.9.3. EV Characterization by NTA

Particle size distribution of isolated EV suspensions was determined by NTA using a NanoSight NS300 system (Malvern Technologies, Malvern, UK) configured with a 488 nm laser and a high-sensitivity scientific CMOS camera as previously described [51]. Isolated EVs were diluted 1:200 with 0.2 µm filtered PBS. The chamber of the NanoSight NS300 was loaded with diluted EVs using a 1 mL syringe. A syringe pump at speed 40 was used to maintain a steady flow rate of EVs during video acquisition. Three 30 s videos were acquired using camera level 14. NTA software version 3.2.16 (Malvern Panalytical) was used to track particles and analyze data using detection threshold 5.

#### 4.9.4. Immunoblotting

For protein analysis, 1× RIPA lysis was added to resuspended EV pellets or EV-treated NHLFs, and the resulting protein was quantified using a Lowry assay as previously described [52]. Protein (50 μg per sample) was boiled for 10 min in solution with sodium dodecyl sulfate (SDS), subjected to sodium dodecyl sulfate polyacrylamide gel electrophoresis (SDS-PAGE) (150 V for 1 h) and transferred onto polyvinylidene difluoride membranes (PVDF; Millipore). Membranes were blocked using 5% skim milk in Tris-buffered saline + 0.1% Tween-20 (TBST). Anti-human primary antibodies were diluted in 5% skim milk in TBST ± 5% BSA and used for immunoblotting as detailed in Supplementary Table S5. Goat anti-mouse IgG and goat anti-rabbit IgG secondary antibodies (Calbiochem, Billerica, MA, USA) conjugated to horseradish peroxidase and diluted in 5% skim milk in TBST ± 5% BSA were used at concentrations of 1:1000. Protein expression was visualized using Amersham ECL Prime Detection Reagent (GE Healthcase, Wauwatosa, WI, USA).

### 4.10. Statistical Analysis

In vitro experiments were performed a minimum of three times with at least three technical replicates included in each experiment. In vivo studies were carried out using multiple mice as detailed in Section 4.2. above. In all cases, quantitative data were compiled from all experiments. Unless otherwise noted, data are presented as the mean ± SEM. Statistical analysis was performed using GraphPad Prism 7.0 software (GraphPad Software, San Diego, CA, USA) using analysis of variance (ANOVA) with Tukey post-tests (for comparison between all media conditions or RNA expression). Values of *p* ≤ 0.05 were considered to be statistically significant.

## 5. Conclusions

In conclusion, the underlying mechanisms that drive differences in metastatic organ tropism between different breast cancer molecular subtypes are unknown. In this study, we reveal for the first time that TN primary tumors in particular have the ability to induce premetastatic changes in both the ECM and soluble components of the lung microenvironment and in distant sites such as the bone marrow, a process that is potentially mediated by breast cancer-derived EVs. These changes are associated with the capacity to develop a “fertile” premetastatic niche in the lung and to support metastatic behaviors such as migration and proliferation that are needed to recruit breast cancer cells from the primary tumor and assist in lung colonization. Notably, these supportive premetastatic changes were not observed in mice bearing luminal A primary tumors, suggesting the possibility of subtype-dependent alterations in the lung microenvironment. Overall, elucidation of how the cancer-induced components of the metastatic niche evolve in relation to prognostic features such as molecular subtype could facilitate improved clinical management of breast cancer patients, with the goal of earlier detection, treatment and/or prevention of lung metastasis in breast cancer patients.

## Figures and Tables

**Table 1:** Metastasis-associated proteins unique to lung-conditioned media (LCM) generated from the lungs of mice bearing triple-negative SUM159 primary tumors (*n* = 3).

Array Position	Protein Name	Function/Association with Metastasis	Reference(s)
60	CCL7	Part of the C–C family and a potent chemoattractant Drives breast cancer proliferation, migration, invasion and EMT Involved in homing breast cancer cells to secondary sites of metastasis Overexpression promotes lung metastasis	[21,22]
124	FGFR4	Receptor for fibroblast growth factors Mediates breast cancer cell proliferation, migration and lung metastasis Increased expression associated with decreased overall survival in patients	[23,24]
149	GM-CSF	Part of the colony stimulating factor family, produced at local sites of inflammation Promotes lung metastasis by the recruitment and mobilization of Ly6G^+^Ly6C^+^ granulocytes for the induction of angiogenesis	[25]
323	MMP3	Part of the MMP family, involved in degrading/regulating the ECM Involved in breast cancer cell invasion, EMT and lung metastasis	[26]
373	Thrombospondin 1	Regulates cellular phenotype and ECM structure Promotes angiogenesis, mediates lung metastasis and promotes breast cancer tumor progression Modulates immunosuppression at secondary sites	[27]
432	VEGF	Exerts angiogenic functions through the activation of VEGFR1 and VEGFR2 Involved in vascular permeability, cancer proliferation and motility Produced by resident lung S100A4^+^ fibroblasts and induces activation of lung endothelial cells during lung metastasis	[28,29]

## Supplementary Materials

The following are available online at https://www.mdpi.com/2072-6694/12/1/172/s1: Figure S1: In vivo primary tumor growth of MCF7 and SUM159 breast cancer cells. Figure S2: Flow cytometry analysis of murine CD117^+^ bone marrow cells in mice bearing MCF7 or SUM159 primary breast tumors. Figure S3: Lung-conditioned media (LCM) generated from the lungs of mice bearing triple-negative SUM159 primary tumors contains metastasis-associated proteins. Figure S4: Characterization of breast cancer-derived extracellular vesicles (EVs). Table S1: Protein Array Analysis of Lung-Conditioned Media (*n* = 3 per group). Table S2: Proteins unique to lung-conditioned media (LCM) generated from the lungs of mice bearing triple-negative SUM159 primary tumors (*n* = 3). Table S3: Antibodies used for immunohistochemistry (IHC). Table S4: TaqMan probes used to evaluate the relative mRNA expression of murine ECM and effector genes in lung tissue or human ECM genes in normal human lung fibroblasts. Table S5: Antibodies used for immunoblotting.
