# Supplementary material for: Triple-Negative Primary Breast Tumors Induce Supportive Premetastatic Changes in the Extracellular Matrix and Soluble Components of the Lung Microenvironment
Source: Cancers (Basel). 2020 Jan 10;12(1):172. doi: 10.3390/cancers12010172 (PMC7016570; doi:10.3390/cancers12010172)
Supplement: Supplementary file 1 [file cancers-12-00172-s001.zip › cancers-677519-suppl-final/cancers-677519-supple-final.docx]

Article

**Triple-Negative Primary Breast Tumors Induce Supportive Premetastatic Changes in the Extracellular Matrix and Soluble Components of the Lung Microenvironment**

Braeden Medeiros, David Goodale, Carl Postenka, Lori E. Lowes, Patti Kiser, Stephen Hearn, Nikki Salmond, Karla C. Williams and Alison L. Allan

Supplementary Materials


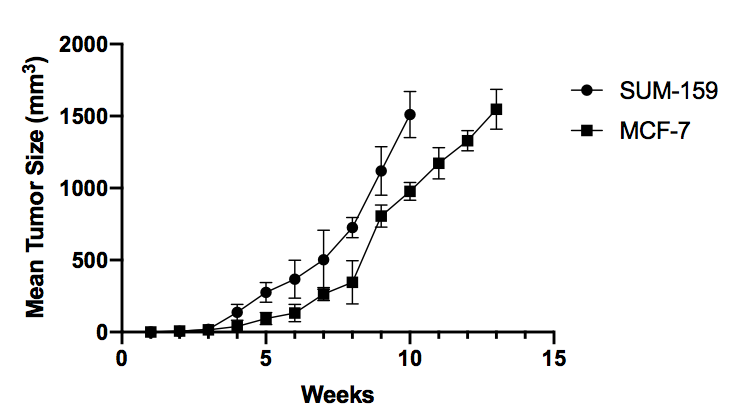


**Supplementary Figure S1.** In vivo primary tumor growth of MCF7 and SUM159 breast cancer cells. MCF7 (luminal A) and SUM159 (TN) human breast cancer cells (1 × 10^6^ cells/mouse) were injected into the mammary fat pad of 6-8 week old female nude mice (*n* = 54 mice/group). Mice injected with MCF7 cells (and a matched tumor-naïve control group) were implanted with subcutaneous time-release estrogen pellets 0.10mg/pellet with 90 days release for the duration of the experiments. Primary tumor size was longitudinally assessed using weekly digital caliper measurements in 2 perpendicular dimensions and calculated using the formula: volume = 0.52 × (width)^2^ × (length). Primary tumors were allowed to grow up to 1500 mm^3^, with mice for tumor-bearing groups (SUM159/MCF7) sacrificed at the same time as age-matched, tumor-naïve control mice. Data are presented mean ± SEM.


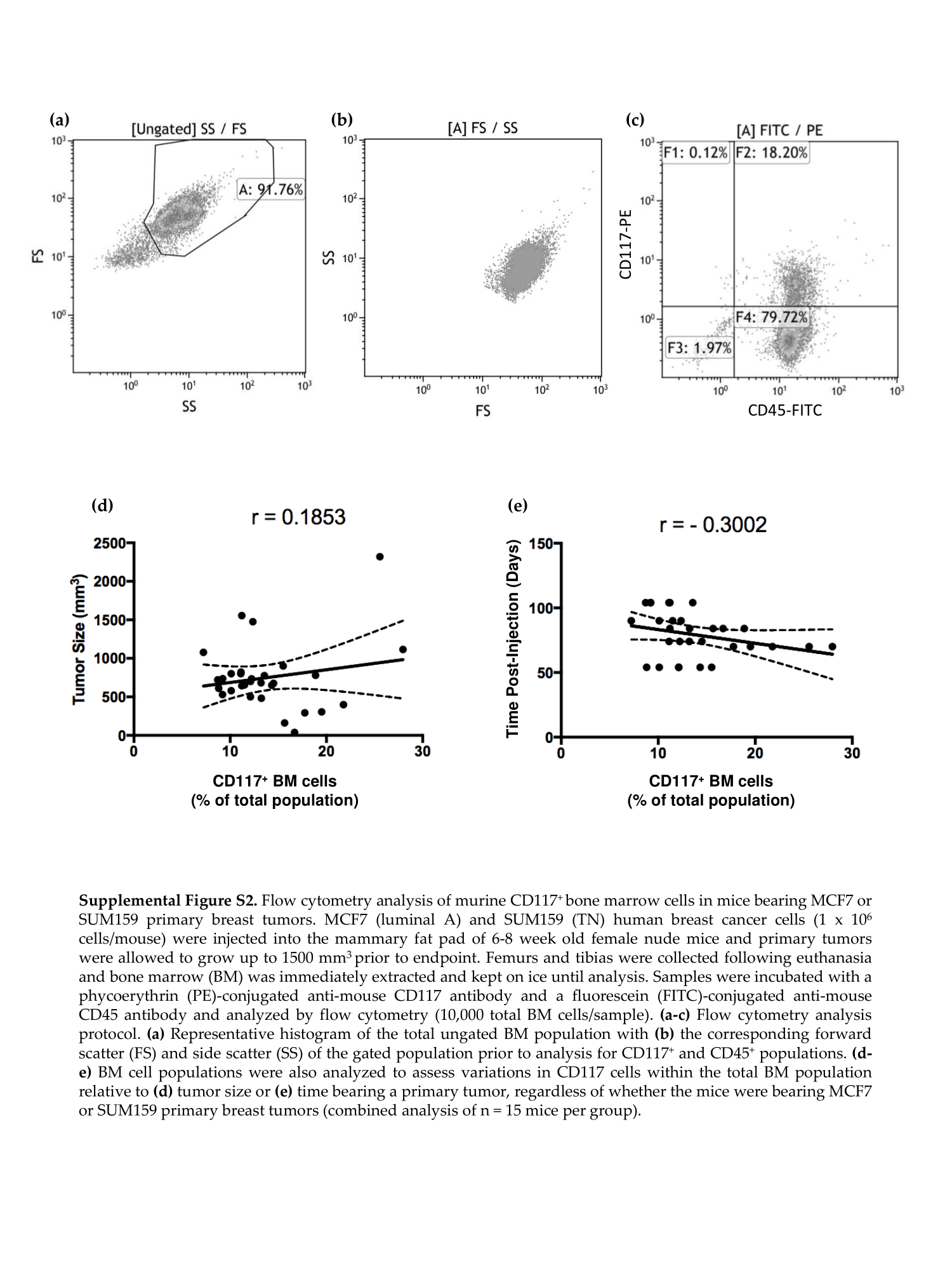


**Supplementary Figure S2.** Flow cytometry analysis of murine CD117^+^ bone marrow cells in mice bearing MCF7 or SUM159 primary breast tumors. MCF7 (luminal A) and SUM159 (TN) human breast cancer cells (1 × 10^6^ cells/mouse) were injected into the mammary fat pad of 6-8 week old female nude mice and primary tumors were allowed to grow up to 1500 mm^3^ prior to endpoint. Femurs and tibias were collected following euthanasia and bone marrow (BM) was immediately extracted and kept on ice until analysis. Samples were incubated with a phycoerythrin (PE)-conjugated anti-mouse CD117 antibody and a fluorescein (FITC)-conjugated anti-mouse CD45 antibody and analyzed by flow cytometry (10,000 total BM cells/sample). (**a**–**c**) Flow cytometry analysis protocol. (**a**) Representative histogram of the total ungated BM population with (**b**) the corresponding forward scatter (FS) and side scatter (SS) of the gated population prior to analysis for CD117+ and CD45+ populations. (**d**,**e**) BM cell populations were also analyzed to assess variations in CD117 cells within the total BM population relative to (**d**) tumor size or (**e**) time bearing a primary tumor, regardless of whether the mice were bearing MCF7 or SUM159 primary breast tumors (combined analysis of *n* = 15 mice per group).


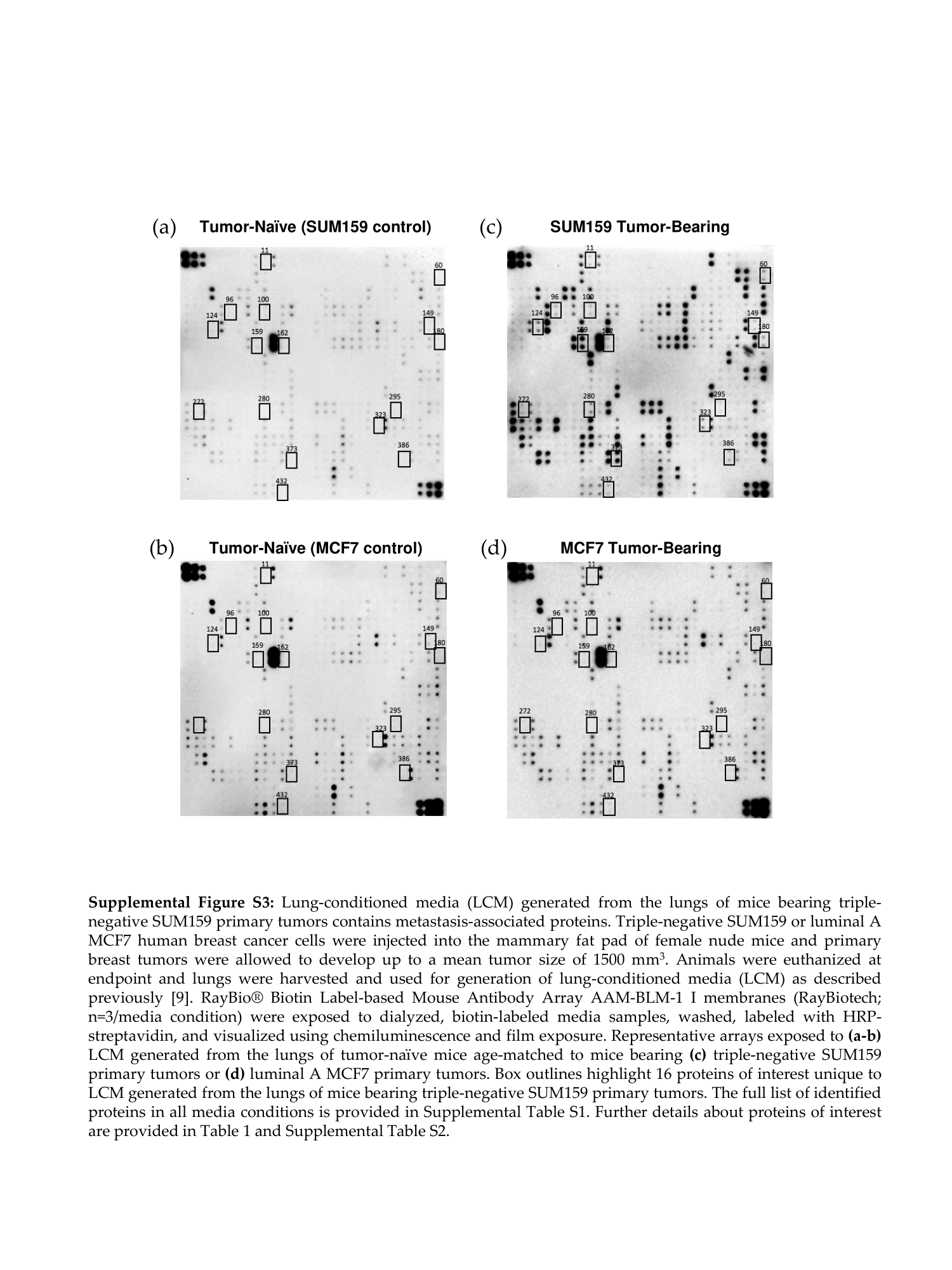


**Supplementary Figure S3.** Lung-conditioned media (LCM) generated from the lungs of mice bearing triple-negative SUM159 primary tumors contains metastasis-associated proteins. Triple-negative SUM159 or luminal A MCF7 human breast cancer cells were injected into the mammary fat pad of female nude mice and primary breast tumors were allowed to develop up to a mean tumor size of 1500 mm^3^. Animals were euthanized at endpoint and lungs were harvested and used for generation of lung-conditioned media (LCM) as described previously [9]. RayBio^®^ Biotin Label-based Mouse Antibody Array AAM-BLM-1 I membranes (RayBiotech; *n* = 3/media condition) were exposed to dialyzed, biotin-labeled media samples, washed, labeled with HRP streptavidin, and visualized using chemiluminescence and film exposure. Representative arrays exposed to (**a**,**b**) LCM generated from the lungs of tumor-naïve mice age-matched to mice bearing (**c**) triple-negative SUM159 primary tumors or (**d**) luminal A MCF7 primary tumors. Box outlines highlight 16 proteins of interest unique to LCM generated from the lungs of mice bearing triple-negative SUM159 primary tumors. The full list of identified proteins in all media conditions is provided in Supplemental Table S1. Further details about proteins of interest are provided in Table 1 and Supplementary Table S2.


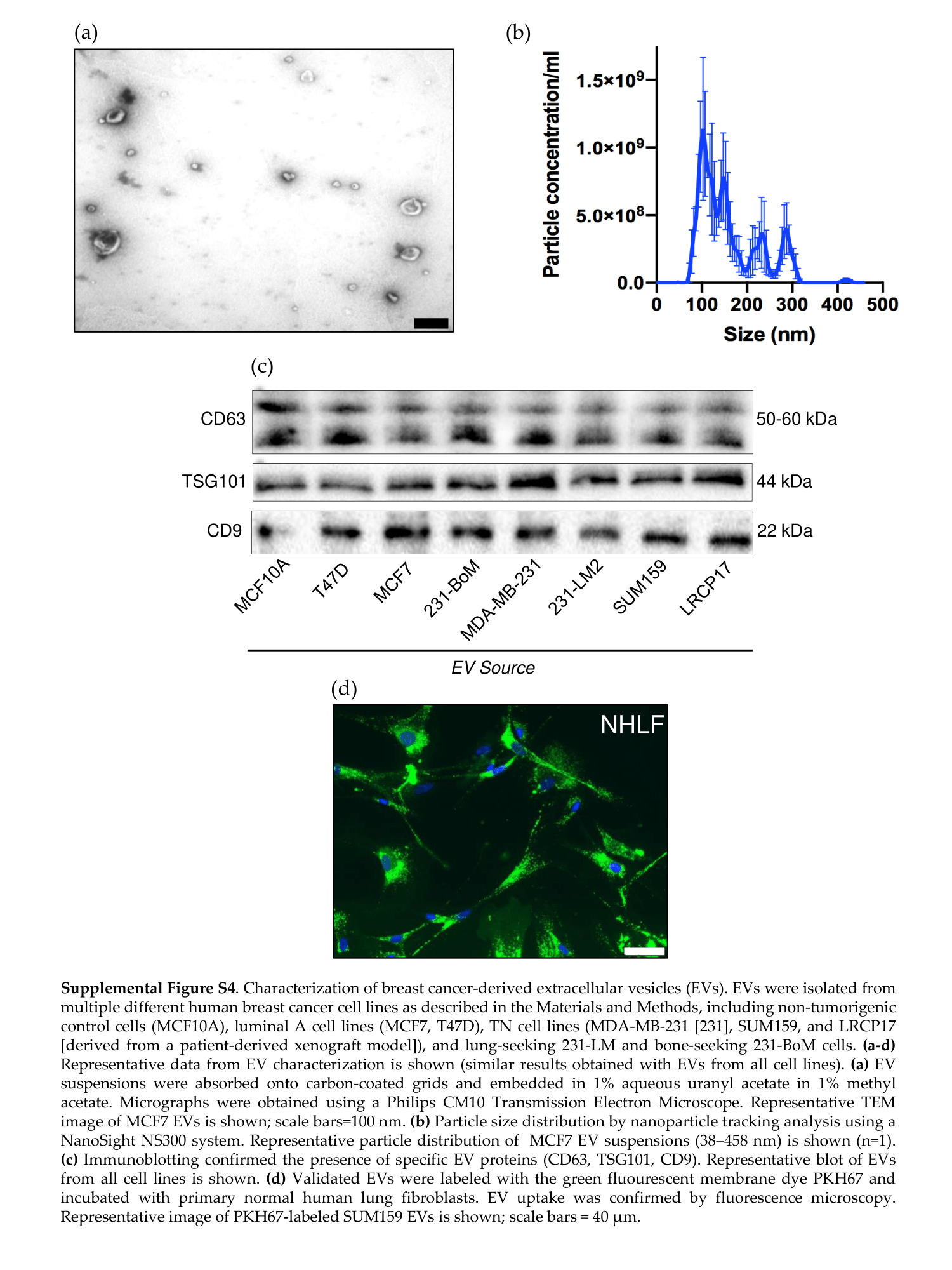


**Supplementary Figure S4.** Characterization of breast cancer-derived extracellular vesicles (EVs). EVs were isolated from multiple different human breast cancer cell lines as described in the Materials and Methods, including non-tumorigenic control cells (MCF10A), luminal A cell lines (MCF7, T47D), TN cell lines (MDA-MB-231 (231), SUM159, and LRCP17 [derived from a patient-derived xenograft model]), and lung-seeking 231-LM and bone-seeking 231-BoM cells. (**a**–**d**) Representative data from EV characterization is shown (similar results obtained with EVs from all cell lines). (**a**) EV suspensions were absorbed onto carbon-coated grids and embedded in 1% aqueous uranyl acetate in 1% methyl acetate. Micrographs were obtained using a Philips CM10 Transmission Electron Microscope. Representative TEM image of MCF7 EVs is shown; scale bars = 100 nm. (**b**) Particle size distribution by nanoparticle tracking analysis using a NanoSight NS300 system. Representative particle distribution of MCF7 EV suspensions (38–458 nm) is shown (*n* = 1). (**c**) Immunoblotting confirmed the presence of specific EV proteins (CD63, TSG101, CD9). Representative blot of EVs from all cell lines is shown. (**d**) Validated EVs were labeled with the green fluourescent membrane dye PKH67 and incubated with primary normal human lung fibroblasts. EV uptake was confirmed by fluorescence microscopy. Representative image of PKH67-labeled SUM159 EVs is shown; scale bars = 40 μm.

**Supplemental Table S2.** Proteins unique to lung-conditioned media (LCM) generated from the lungs of mice bearing triple-negative SUM159 primary tumors (*n* = 3).

| **Array Position** | **Protein Name** |
| --- | --- |
| 11 | Activin C |
| 60 | CCL7 |
| 96 | DAN |
| 100 | Dkk-4 |
| 124 | FGF R4 |
| 149 | GM-CSF |
| 159 | ICAM-2 |
| 162 | IFN-alpha/beta R1 |
| 180 | IL-1 R6 |
| 272 | IL-17RC |
| 280 | IL-22BP |
| 295 | Kremin-2 |
| 323 | MMP3 |
| 373 | Thrombospondin-1 |
| 386 | TMEF1 |
| 432 | VEGF |

**Supplemental Table S3.** Antibodies used for immunohistochemistry (IHC).

| **Antigen** | **Clone** | **Dilution** | **Source** |
| --- | --- | --- | --- |
| Anti-Human Mitochondrial Cytochrome C Oxidase | MTCO2 | 1/100 | Invitrogen |
| Anti-Mouse Fibronectin | F14 | 1/5000 | Abcam |
| Anti-Mouse Periostin | EPR20806 | 1/6000 | Abcam |
| Anti-Mouse Tenascin-C | EPR4219 | 1/4000 | Abcam |
| Anti-Mouse MMP9 |  | 1/5000 | Abcam |
| Anti-Mouse MMP9 | 56-2A4 | 1/5000 | Abcam |

**Supplementary Table S4.** TaqMan probes used to evaluate the relative mRNA expression of murine ECM and effector genes in lung tissue or human ECM genes in normal human lung fibroblasts.

| **Gene Target** | **Taqman Assay ID #** | **Distributor** |
| --- | --- | --- |
| Murine fibronectin | Mm01256744_m1 | ThermoFisher |
| Murine periostin | Mm00495386_m1 | ThermoFisher |
| Murine tenascin-c | Mm00495662_m1 | ThermoFisher |
| Murine collagen A1 | Mm00801666_g1 | ThermoFisher |
| Murine MMP9 | Mm00442991_m1 | ThermoFisher |
| Murine LOX | Mm01284919_m1 | ThermoFisher |
| Murine CCL2 | Mm00441242_m1 | ThermoFisher |
| Murine GAPDH | Mm99999915_g1 | ThermoFisher |
| Human fibronectin | Hs01549976_m1 | ThermoFisher |
| Human periostin | Hs01566750_m1 | ThermoFisher |
| Human GAPDH | Hs02786624_g1 | ThermoFisher |

**Supplementary Table S5.** Antibodies used for immunoblotting.

| **Antigen** | **Clone** | **Dilution** | **Source** |
| --- | --- | --- | --- |
| Anti-human CD63 | EPR21151 | 1/1000 | Abcam |
| Anti-Human TSG101 | 4A10 | 1/1000 | Abcam |
| Anti-Human CD9 | EPR2949 | 1/2000 | Abcam |
| Anti-Human Fibronectin | F1 | 1/1500 | Abcam |
| Anti-Human Periostin | EPR6989(N)(B) | 1/500 | Abcam |
| Anti-Human β-Actin | AC-74 | 1/1000 | Sigma-Aldrich |

| 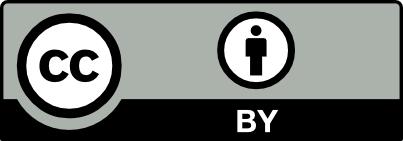 | © 2020 by the authors. Licensee MDPI, Basel, Switzerland. This article is an open access article distributed under the terms and conditions of the Creative Commons Attribution (CC BY) license (http://creativecommons.org/licenses/by/4.0/). |
| --- | --- |
