# Supplementary material for: Triple-Negative Primary Breast Tumors Induce Supportive Premetastatic Changes in the Extracellular Matrix and Soluble Components of the Lung Microenvironment
Source: Cancers (Basel). 2020 Jan 10;12(1):172. doi: 10.3390/cancers12010172 (PMC7016570; doi:10.3390/cancers12010172)
Supplement: Supplementary file 1 [file cancers-12-00172-s001.zip › cancers-677519-suppl-final/cancers-677519-western blot figures.pdf]

Supplemental Materials

Triple-negative primary breast tumors induce supportive premetastatic changes in the soluble and extracellular matrix components of the lung microenvironment

Braeden Medeiros, David Goodale, Carl Postenka, Lori Lowes, Patti Kiser, Stephen Hearn, Nikki Salmond, Karla C. Williams and Alison L. Allan

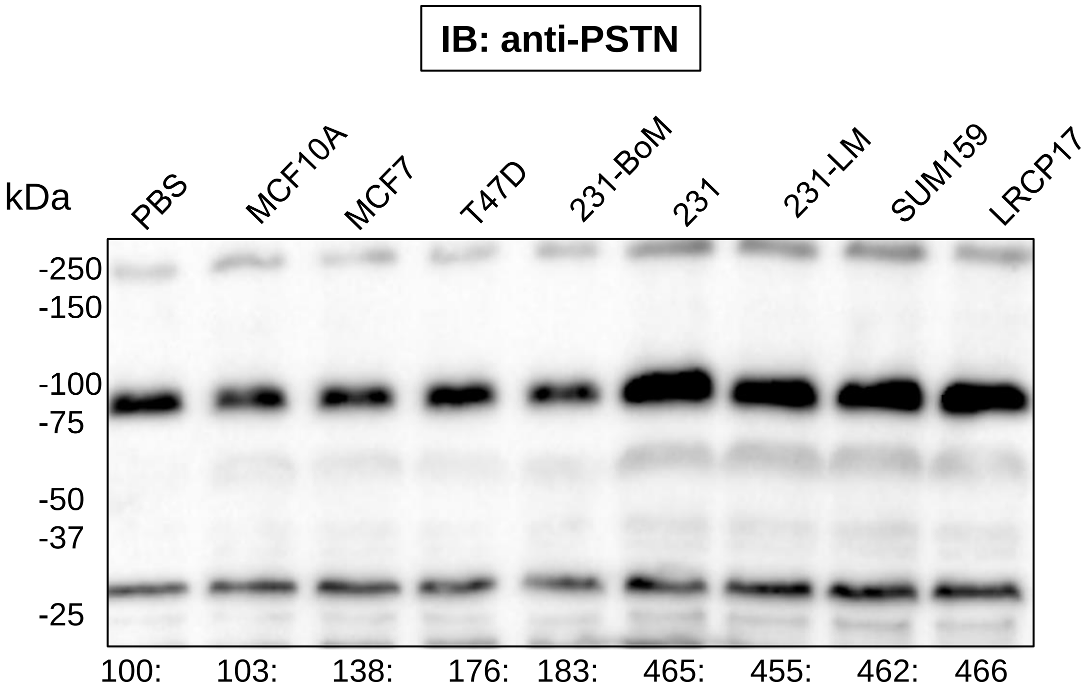

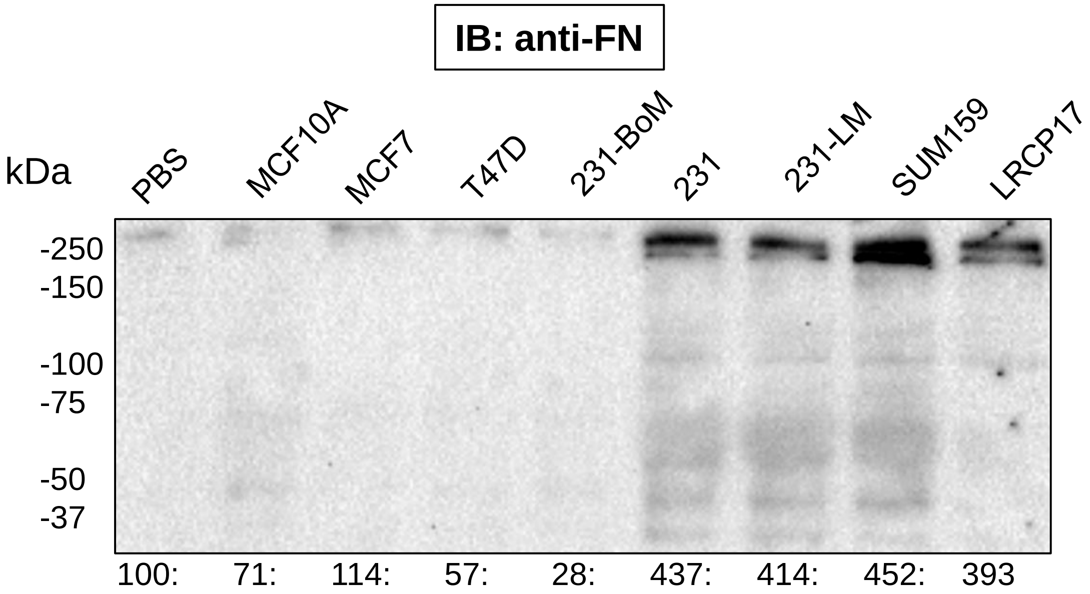

Supplemental Materials SM1. Full representative immunoblots showing protein expression, molecular weight, and densitometry values of periostin (PSTN) and fibronectin (FN) in normal human fibroblasts treated with breast cancer-derived extracellular vesicles (EVs). PBS was set as the normalization control (value of 100 for each individual blot). Cropped immunoblots and compiled quantitative densitometry analysis (n=3) are presented in Figure 6.

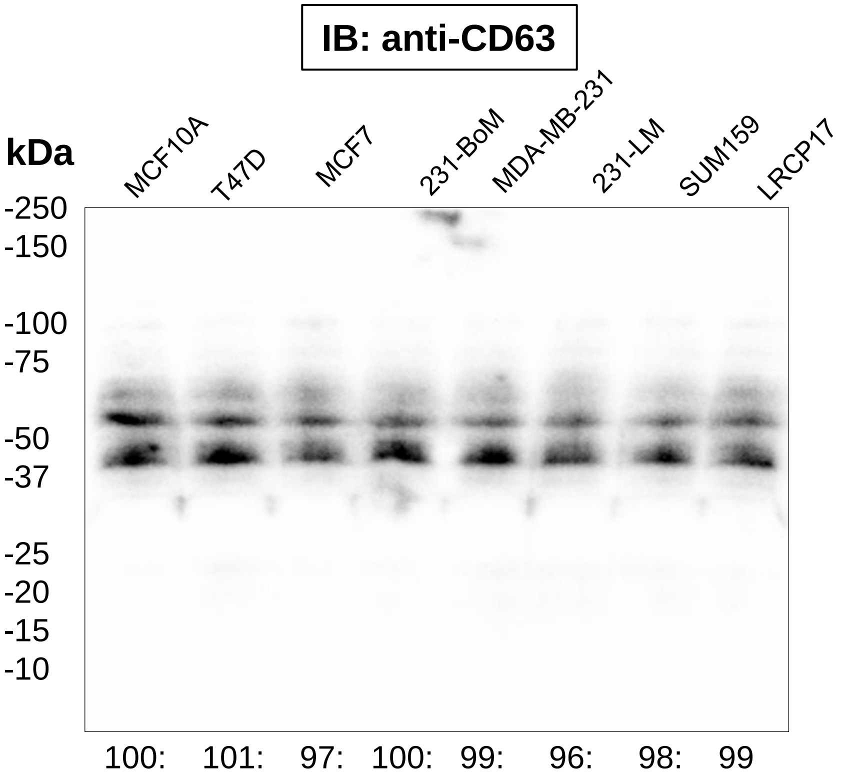

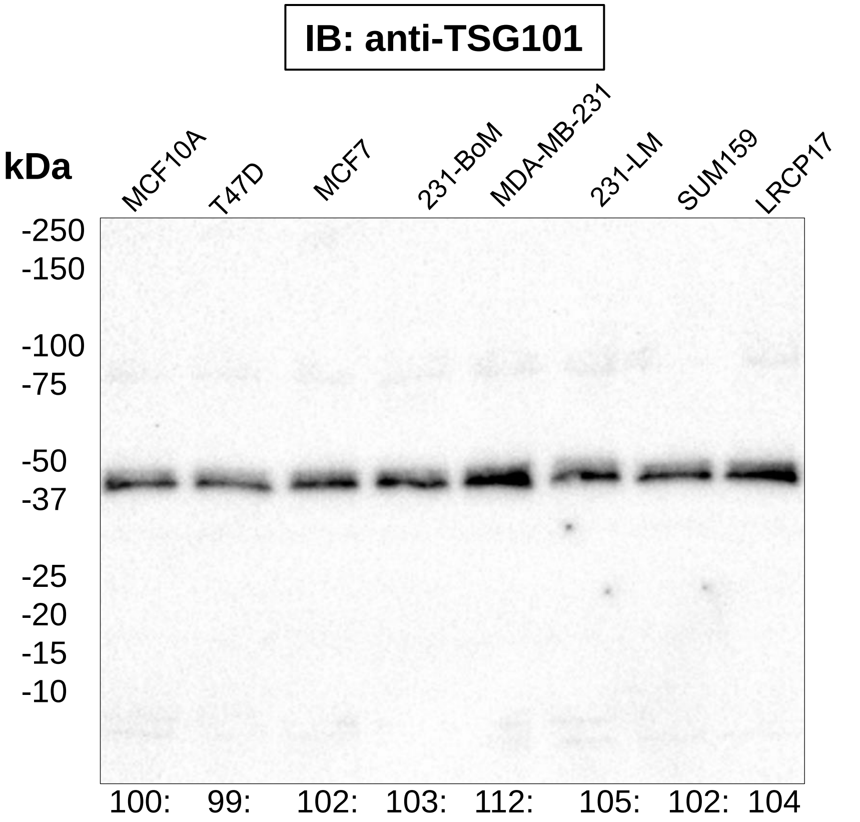

Supplemental Materials SM2. Full representative immunoblots showing protein expression and molecular weight of CD63 and TSG101 in breast cancer-derived extracellular vesicles (EVs). Cropped immunoblots are presented in Supplemental Figure S4. MCF10A was set as the normalization control (value of 100 for each individual blot).

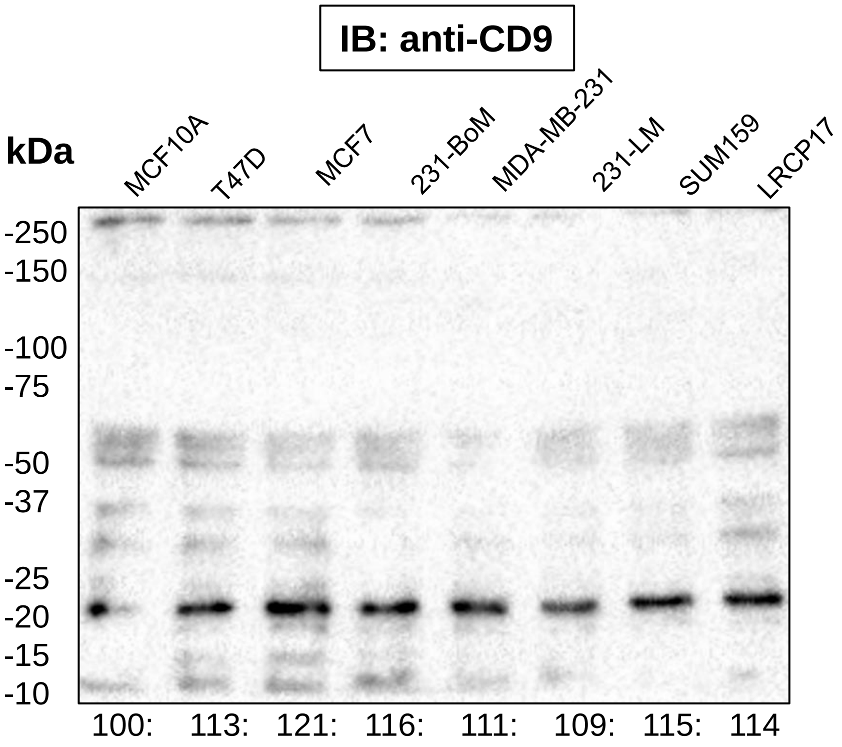

Supplemental Materials SM3. Full representative immunoblots showing protein expression and molecular weight of CD9 in breast cancer-derived extracellular vesicles (EVs). Cropped immunoblots are presented in Supplemental Figure S4. MCF10A was set as the normalization control (value of 100 for each individual blot).
